# Potentials of Low-Budget Microdrones: Processing 3D Point Clouds and Images for Representing Post-Industrial Landmarks in Immersive Virtual Environments

**DOI:** 10.3389/frobt.2022.886240

**Published:** 2022-05-24

**Authors:** Marco Weißmann, Dennis Edler, Andreas Rienow

**Affiliations:** Geomatics Group, Institute of Geography, Ruhr-University Bochum (RUB), Bochum, Germany

**Keywords:** microdrones, UAV, 3D mapping, point clouds, virtual reality

## Abstract

Post-industrial areas in Europe, such as the Rhine-Ruhr Metropolitan region in Germany, include cultural heritage sites fostering local and regional identities with the industrial past. Today, these landmarks are popular places of interest for visitors. In addition to portable camera devices, low-budget ultra-lightweight unmanned aerial vehicles, such as micro quadcopter drones, are on their way to being established as mass photography equipment. This low-cost hardware is not only useful for recreational usage but also supports individualized remote sensing with optical images and facilitates the acquisition of 3D point clouds of the targeted object(s). Both data sets are valuable and accurate geospatial data resources for further processing of textured 3D models. To experience these 3D models in a timely way, these 3D visualizations can directly be imported into game engines. They can be extended with modern interaction techniques and additional (semantic) information. The visualization of the data can be explored in immersive virtual environments, which allows, for instance, urban planners to use low-cost microdrones to 3D map the human impact on the environment and preserve this status in a 3D model that can be analyzed and explored in following steps. A case example of the old wage hall of the Zeche “Bonifacius” (Essen, Germany) with its simple building structure showed that it is possible to generate a detailed and accurate 3D model based on the microdrone data. The point cloud which the 3D model of the old wage hall was based on represented partly better data accuracy than the point clouds derived from airborne laser scanning and offered by public agencies as open data. On average, the distance between the point clouds was 0.7 m, while the average distance between the airborne laser scanning point cloud and the 3D model was −0.02 m. Matching high-quality textures of the building facades brings in a new aspect of 3D data quality which can be adopted when creating immersive virtual environments using the Unity engine. The example of the wage hall makes it clear that the use of low-cost drones and the subsequent data processing can result in valuable sources of point clouds and textured 3D models.

## 1 Introduction

The metropolitan region Rhine–Ruhr is known for its cultural heritage related to old industrial times (Müller and Carr 2009; Otto 2019). After manufacturing and, especially, coal mining decreased, brownfields, (often contaminated) mining heaps, and sites were revitalized. In the course of large structural change initiatives in the Ruhr area, many formerly industrial sites became destinations for recreational activities, which led to new forms of human–nature interactions in this region (Kilper 1999). Industrial urbanity becomes idealized and revitalized urban sites of industrial times shape out a new symbology of a “wild nature” (Kühne 2019). These sites are often considered as “landmarks” of cultural heritage (Edler et al., 2019).

Visiting these landmarks, such as old mining sites, attracts citizens. Such visits are accompanied by the usage of technologies to store impressions, such as photography. The modern technology of photography not only includes handheld camera devices but also microdrones which can be considered as affordable mass media devices. They can be used for individualized flights around areas of interest, such as touristic sites, and their media output goes beyond 2D images. These microdrones facilitate the acquisition of 3D point cloud data coupled with 2D images that could be used to derive matching 3D textures (Carvajal-Ramírez et al., 2019; Neitzel and Klonowski 2011; Nex and Remondino 2014). Based on established processing steps and modern software, this microdrone-based data collection can lead to photorealistic 3D models that can be imported into computer game engines and then be visualized and experienced in immersive virtual environments.

Different studies exist which are methodologically built on microdrones and structure-from-motion (SfM) approaches (e.g., Mora et al., 2019; Aboutalebi et al., 2019; Kwon et al., 2017). Mora et al. (2019) used a DJI Phantom 4 Pro apparatus. They examined the accuracy of an sUAS-derived point cloud of a parking lot located in Ontario, California, by comparing it to ground control points (GCPs). Aboutalebi et al. (2019) proposed a methodology used to incorporate 3D information extracted from an UAV point into a two-source energy balance (TSEB) model. Their drone AggieAir was developed by Utah State University. Kwon et al. (2017) used the potentials of a DJI Phantom 3 Advanced apparatus to develop a hybrid scanning method for the 3D modeling and analysis of atypical ground shapes which are continually changing according to the construction situation. These studies point to the diversity of sensors, methodologies, and applications. As recently stated by Ventura et al. (2022), with reference to an application of underwater monitoring, “no standardized methods currently exist to provide 3-dimensional high spatial resolution and accuracy cartographic products” (Balletti et al., 2015). Moreover, the potentials for exploring the strengths and usefulness of UAS data acquired through low-budget equipment are considered high (Papakonstantinou et al., 2019). This lack of established standard methods which are also valid for the 3D investigation and visualization of cultural heritage sites calls for an exploration of new sensors and methodologies.

In an exploratory research approach, we investigated the potentials but also the limitations of the data accuracy acquired with a low-budget microdrone (DJI Mavic Mini, 249 g). To consider a typical postindustrial colliery site in the Ruhr Area (“Zeche Bonifacius” in Essen), and, methodologically speaking, to conduct a more differentiated analysis, we selected a case example that contains a post-industrial wage hall (used as a hotel today). As an example of citizen science, the applied method provides the possibility for experts and non-experts to generate and evaluate 3D reconstructions using a customary (low-budget) ultralight and foldable drone.

In this study, we 1) present a methodological workflow of how to create an immersive virtual environment from low-budget microdrone data based on processing steps conducted by open-source software. Moreover, we 2) discuss the data accuracy referring to the wage hall in our case example and in comparison to other openly available (geospatial) data sources. The key question of this exploratory approach is: how do the point clouds generated by the low-budget microdrone perform in comparison to airborne laser-scanning (ALS) point clouds provided by governmental surveying departments as open geospatial data sets? The performance analysis includes the established 3D deviation measures of the cloud-to-cloud distance and cloud-to-mesh distance (Esposito et al., 2017; Sofonia et al., 2019).

In terms of the methodological workflow, the underlying processing steps of the OpenDroneMap software for the derivation of point clouds and 3D models are based on the following individual freely available software components. The point cloud is created using the overlapping images from the drone using a structure-from-motion method. The point cloud is further processed into a polygon mesh using the Screened Poisson surface reconstruction algorithm. Based on the input images, this polygon mesh is textured by Mvs-Texturing software. The result is a textured 3D model that is available in the open file format Wavefront OBJ file (.obj) and can be imported in Unity and other software applications compatible with the file format.

Section 2 describes the hardware and software used to acquire images and process them in order to create point clouds and 3D models. In addition, the description of the workflow explains how to evaluate the created point clouds and 3D models using the cloud-to-cloud distance and cloud-to-mesh distance. The results of the evaluation are presented in Section 3 and are then discussed in Section 4. Finally, Section 5 summarizes the results and provides an outlook on further research topics.

## 2 Case Study, Materials, and Methods (From Unmanned Aerial Vehicle Images to Immersive Virtual Environments)

**FIGURE 1:**
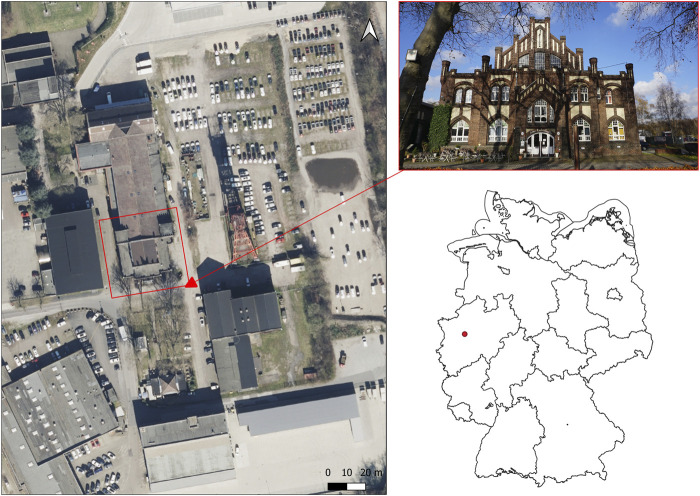
Location of the former colliery site in Essen-Kray [digital orthophotos from the district government of Cologne and the administrative boundaries of Germany from GeoBasis-DE/BKG (2022)].

The specified study object (case study) is located on the site of the former Bonifacius colliery. The site is located in the Kray district of the city of Essen. The mine was established in 1851 (Historisches Portal Essen, 2022). Since 1985, the colliery has been under monument protection. The wage hall has a neo-Gothic style facade and is now used as a restaurant and hotel (Regionalverband Ruhr 2021). Figure 1 shows the study site and the wage hall. The wage hall is visually recognizable by dark bricks. In addition, there is plaster on the facades and white window elements. The words “Alte Lohnhalle” (old wage hall) can be read above the entrance area. Today the Bonifacius colliery is part of the Industrial Heritage Trail (Regionalverband Ruhr 2021).

The drone model used for data collection was the DJI Mavic Mini model. This is a drone model for private usage and has a weight of 249 g. The camera sensor is a 1/2.3″ CMOS with a resolution of 12 megapixels (DJI 2019). The camera is mounted on a gimbal, which allows setting the tilt angles of the camera from −90° to +20°. On the bottom side of the microdrone are downward facing vision and infrared sensors (DJI 2019). Obstacle detection is not implemented in this model. The microdrone is controlled *via* remote control. A smartphone is attached to this remote control, which serves as a screen for the manufacturer’s software. The frequencies for signal transmission between the microdrone and controller are 2.4 and 5.8 GHz (DJI 2019), respectively. The used software for the control of the microdrone was the DJI Fly v.1.2.1. The user interface of the software allows flight settings to be made and basic flight information to be read during and before the flight. The drone and associated software were not modified. They were used according to their state of delivery.

### 2.1 Workflow (Low-Budget Microdrone Images to the 3D-Model and Point Clouds)

The workflow applied consists of three parts. First, images of the examination object were acquired with the microdrone. No ground control points were used during data collection, and the georeferencing of imagery collected by the drone was based on GPS coordinates available in the imagery metadata. The control of the drone and the operation of the camera trigger were performed manually. Before each shot, the drone was stopped, and then, the shot was taken while hovering. This was performed to prevent blurry images. The flight altitudes were set *via* the software. The collected images were then processed with the free software OpenDroneMap. This software reconstructs point clouds and textured 3D models of the survey objects from the collected images. For the analysis of the accuracy of the point clouds and 3D models, CloudCompare software was used.

### 2.2 Data Acquisition During Flight Campaign

**FIGURE 2:**
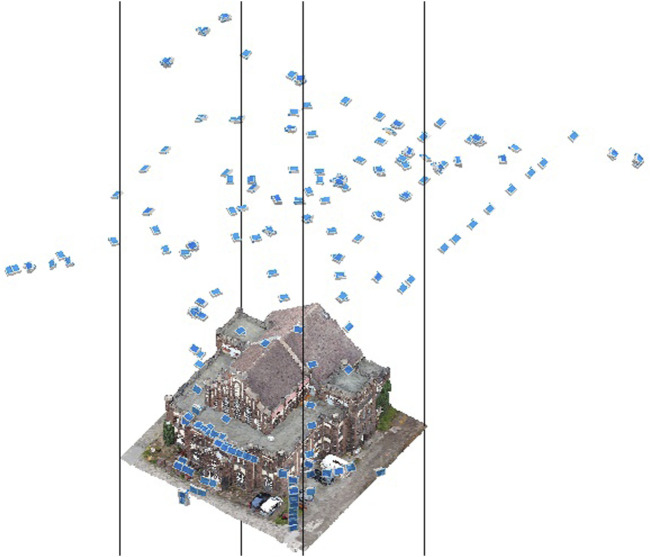
Camera locations of the flight campaign.

The aerial survey of the wage hall was conducted on 26 November 2020 at 11:01 a.m. The data acquisition resulted in 382 images. It was divided into four individual flight sections, each using a charged battery. The camera locations are shown in Figure 2. The flight lasted 66 min from the first to the last image. During the aerial survey, no shadows were visible in the study area due to the presence of cloud cover, which provided a diffuse illumination of the study area. There was no wind during data collection, and the DJI app did not display any warnings for increased wind speeds. During the flight, care was taken to include a high number of oblique images of the building facade to test how detailed the facade could be reconstructed and textured.

### 2.3 Image Data Processing and Point Cloud Preparation

In the next step, the collected images of the wage hall were converted to a point cloud and a textured 3D model using the freely available software WebODM, which is a web interface of the OpenDroneMap software. The collected images were processed in WebODM with the setting of high resolution.

Prior to the analysis, the point clouds from the drone and the 3D measurement data were cut to a uniform study area since the focus of the analysis was placed on the wage hall. In the further course of this work, these point cloud data will be referred to as ALS (airborne laser scanning) point cloud, whereas the point cloud generated from the microdrone data will be named as the UAV (unmanned aerial vehicle) point cloud. The point clouds of the 3D measurement data of the district government of Cologne are available in the form of tiles on a communal scale. The area of the Bonifacius colliery was covered by the data tile with the designation 3dm_32_366_5704_1_nw. It had a total number of 14,772,007 points. For comparison with the UAV point clouds, both data sets should be limited to the study object. This was to prevent the analysis methods from being affected by other objects in the study area. To achieve this processing step, the point clouds were adjusted in the CloudCompare software. In the first processing step, a vector file in the shapefile format was created in the geoinformation software QGIS, covering the area of the old wage hall.

These vector files serve as a template for cropping the point clouds in CloudCompare. The coordinate system of both vector files was transformed to EPSG:32632. At this point, it should be added that the coordinate system of the 3D measurement data from the Cologne district government, which determines the location of the point cloud, was set as EPSG:25832. In preparation for cropping the point clouds from this data source, the vector data were transformed into this coordinate system. Afterward, the point clouds were trimmed to fit the study area.

Both point clouds were aligned to calculate the cloud-to-cloud distance. Weinmann (2016) refers to these processing steps as point cloud registration, point set registration, or 3D scan matching. The process of registration of the two point clouds follows in most cases a first rough approximation, which is then completed by precise matching (Weinmann 2016). For the implementation of the registration, the fine registration function in CloudCompare was used. It is based on the iterative closest point algorithm (CloudCompare 2015). The algorithm is used to align 3D models based on their geometric relation to each other (Rusinkiewicz and Levoy 2001).

The evaluation accuracy of the 3D models reconstructed by the software was also performed with the ALS point clouds. For this purpose, the cloud-to-mesh distance function was chosen. In this method, the distance between the point cloud and the polygon mesh is calculated (CloudCompare 2015). For this step, the data points representing the ground below the wage hall were removed from the ALS point cloud. Since the polygon mesh did not cover this area, these areas were removed from the clipped point cloud so that these areas would not affect the evaluation. The ground areas within the dataset were classified with a value of 2 (Köln 2020a). The remaining points were classified with a value of 3,032. To separate the two classes, the filter-by-value function was used in CloudCompare. The ground values were thus removed from the point cloud. In addition, data points that could be assigned to a tree that was located in front of the pay hall were also removed. These points were removed using the segmentation function. After this processing step, the ALS point cloud had 15,833 points.

### 2.4 Evaluation of the Point Cloud Data Accuracy

For the analysis of the accuracy, the point cloud (.las) created by the software was used. It was compared with the freely available 3D measurement data published by the Cologne district government, which were acquired by airborne laser scanning (Köln 2020b). These datasets were available as point clouds in the file format LAS (.laz). The accuracy of the position is given with a deviation of +/− 30 cm and that of the height with +/− 15 cm (double standard deviation). First, there is the description procedure of the evaluation of the point clouds. Subsequently, the evaluation of the polygon meshes based on the ALS point cloud is explained.

For the analysis of the point cloud properties, the surface density of the point clouds was calculated. In this analysis approach, CloudCompare calculates the number of neighboring points from each point in a predefined radius and stores this number (CloudCompare 2015). The values of 1, 0.3, and 0.15 m were selected for the search radii used to calculate the surface density. In addition, the point density per square meter was calculated by dividing the number of points by the size of the comparison area. The point density of point clouds is an important influencing factor for the reconstruction of surfaces since they represent the surface in the shape of points, and the reconstruction is calculated on the basis of these points (Berger et al., 2017).

**FIGURE 3:**
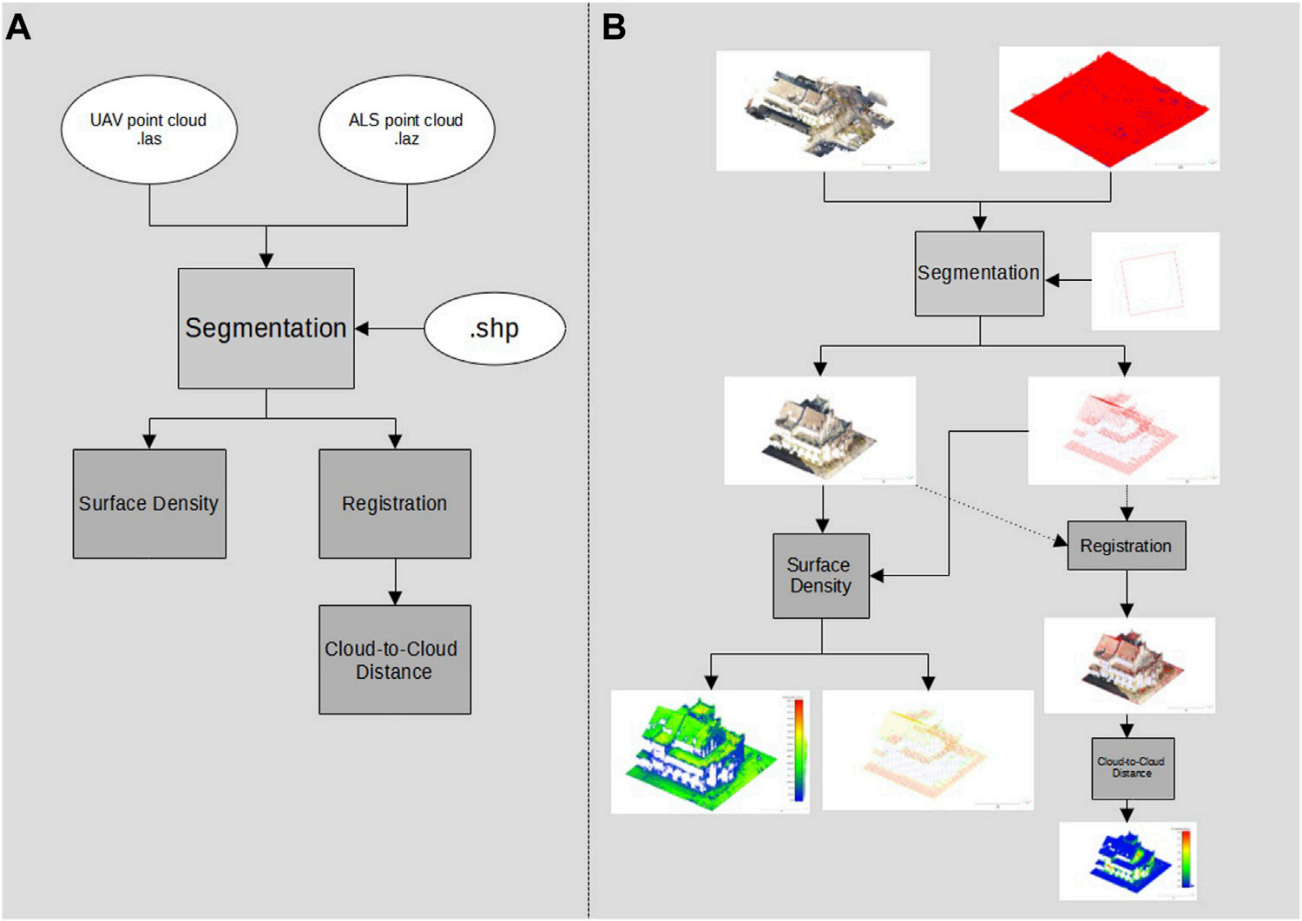
Workflow of the point cloud evaluation (the cloud-to-cloud distance)—overview of data processing steps and formats **(A)** and the illustrated workflow output referring to the case study **(B)**.

As a further analysis approach, the difference between the UAV point cloud and the ALS point cloud has been calculated. This could be implemented with the cloud-to-cloud distance function. This function calculated the Euclidean distance between points of a reference point cloud to the comparison point cloud (CloudCompare 2015). The ALS point cloud was used as the reference point cloud and compared with the UAV point clouds. It should be noted here that inaccuracies can occur if the reference point cloud has a lower density than the point cloud used for the comparison (CloudCompare 2015). A summary of the workflow used to evaluate the data accuracy of the point clouds, along with the previous data preparation, is presented in Figure 3.

### 2.5 Evaluation of the 3D Reconstruction

**FIGURE 4:**
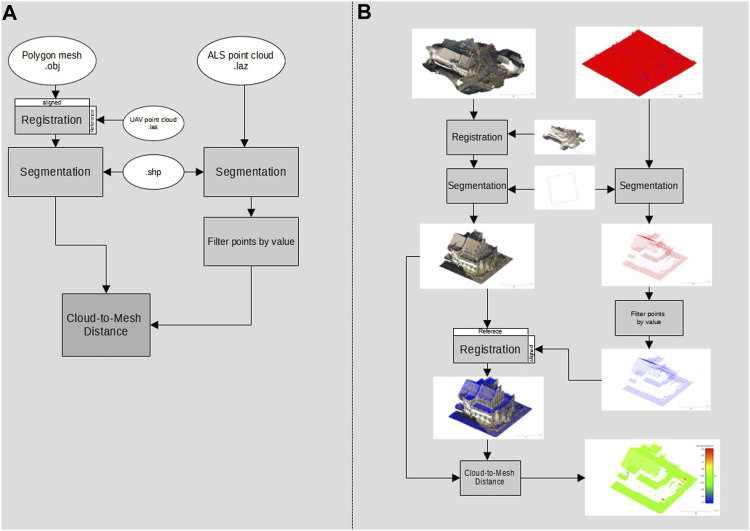
Workflow for the evaluation of the generated 3D model (the cloud-to-mesh distance)—overview of data processing steps and formats **(A)** and the illustrated workflow output referring to the case study **(B)**.

The evaluation of the accuracy of the 3D reconstruction was performed based on the polygon mesh generated in the Wavefront OBJ (.obj) data format by WebODM. Here, the ALS point cloud again served as the comparison data set. As a method for evaluating the accuracy, the cloud-to-mesh distance was calculated. In doing so, the software application calculates the distance of each point of the comparison point cloud to the nearest part of the polygon mesh of the 3D reconstruction. (CloudCompare 2015). Figure 4 shows the workflow performed, from data preparation to the cloud-to-mesh distance.

## 3 Results

According to the main research question dealing with the comparability of point clouds generated by UAV to freely available point clouds generated by ALS, the result presentation is divided into three parts. First, the description of the point cloud properties, in terms of surface density and the number of points, is described. Then, the accuracy, measured by the distance between the UAV and ALS point cloud, is presented based on the cloud-to-cloud distance. Last, the accuracy of the 3D reconstruction of the old wage hall is shown in comparison to the ALS point cloud based on the cloud-to-mesh distance.

### 3.1 Comparison of Point Cloud Properties

**FIGURE 5:**
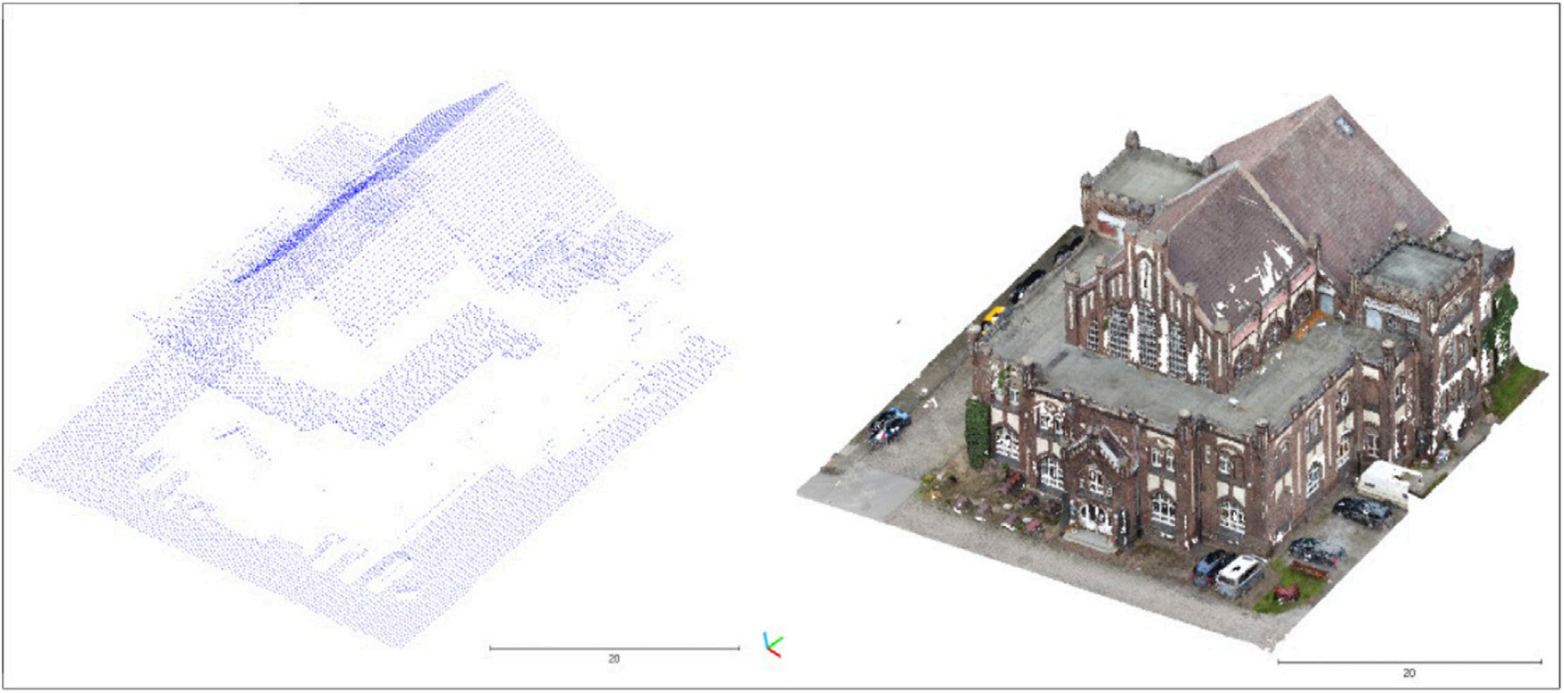
ALS point cloud (left) and UAV point cloud (right).

Table 1 shows the properties of the point clouds and includes the number of points in the entire data set, the number of points in the study area, the surface density, and the number of points per square meter. The entire data tile of the point cloud has a number of 14,722,007 points. After it was cropped to the vector file, there were 16,881 points. This results in a point density of 9.68 points per square meter for the comparison area of the wage hall. The surface density at a radius of 1 m (r = 1) was 7.23 points, with a standard deviation of 2.42 points. At a radius of 0.3 m (r = 0.3), the surface density was 4.57 points and a standard deviation of 1.73 points. For the radius of 0.15 m (r = 0.15), there was a surface density of 14.15 points and a standard deviation of 0.3 points. The coverage of the facades of the wage hall by data points in the ALS point cloud was barely visible when viewed visually, which can be seen in Figure 5. On the east side of the wage hall, data points were visible in the bay window area. In the visual comparison, the increased number of data points of the UAV point cloud can be seen in the area of the building facades. The properties of the UAV point cloud are now described.

**TABLE 1:** Comparison of the point cloud properties of the wage hall.

Point cloud	Total points (whole dataset)	Total points in the study area	Surface density μ	Surface density σ	Points/m2
ALS	14,722,077	16,881	7,23 (r = 1)	2,42 (r = 1)	9.69
			4,57 (r = 0.3)	1,73 (r = 0.3)	
			14,15 (r = 0.15)	0,3 (r = 0.15)	
UAV	21,392,351	9,912,569	4,363.66 (r = 1)	2,120.01 (r = 1)	5,687.07
			4,306.51 (r = 0.3)	2,169.06 (r = 0.3)	
			4,161.29 (r = 0.15)	2,238.01 (r = 0.15)	

**FIGURE 6:**
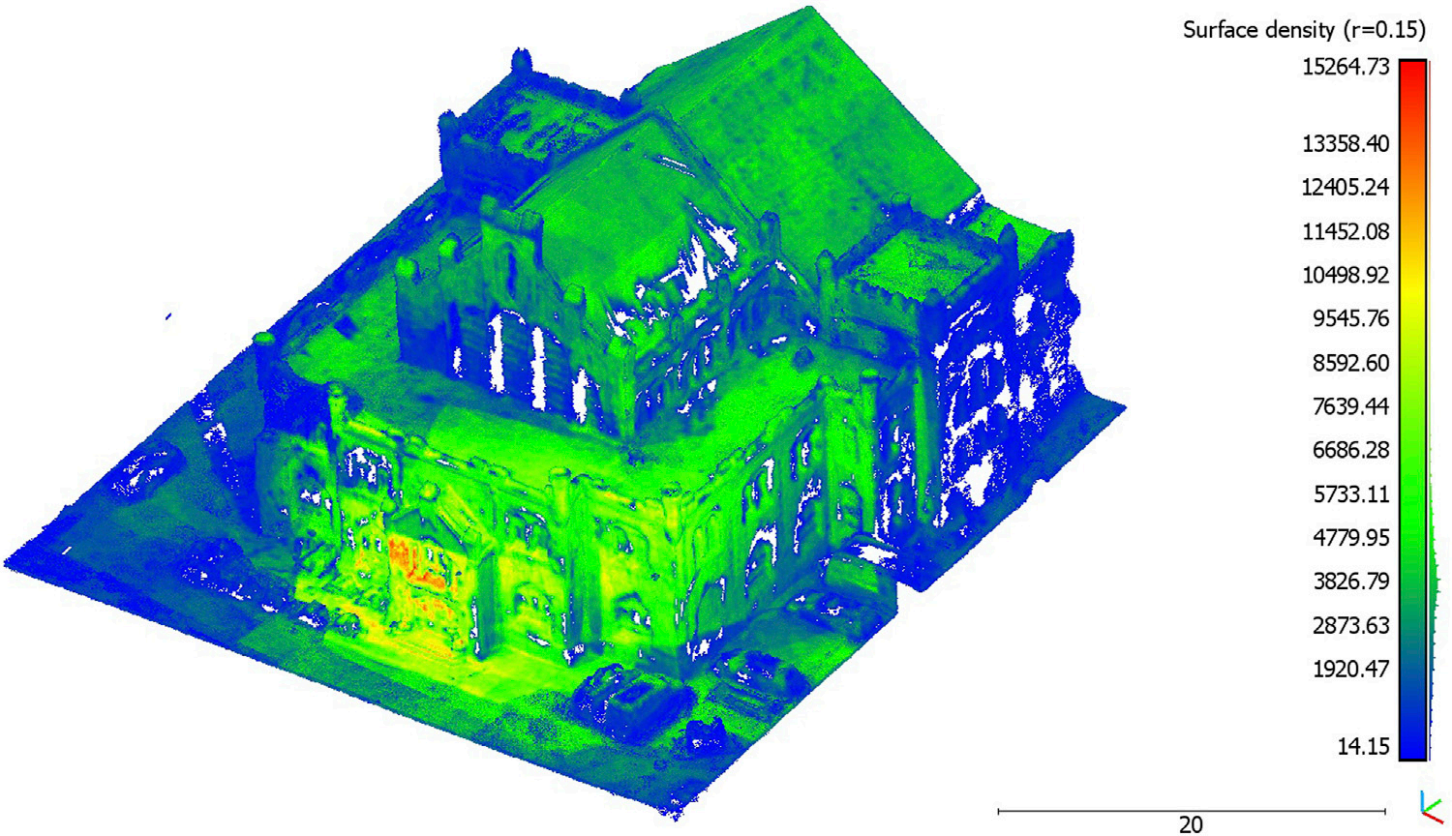
Visualized surface density (r = 0.15)

The point cloud of the UAV-based data collection of the wage hall had a total number of 21,392,351 points. After it was post-processed (cut) to fit the study area, it had a total number of 9,912,569 points. This resulted in a point density of 5,687.07 points per square meter. The mean surface density at a radius of 1 m (r = 1) was 4,363.66 points and had a standard deviation of 2,120.01 points. At a radius of 0.3 m (r = 0.3), the mean surface density was 4306.51 points. The standard deviation was 2,169.06 points. At a radius of 0.15 m (r = 0.15), the mean surface density was 4,161.29 points and had a standard deviation of 2,238.01 points. When considering the visualized surface density (r = 0.15) in Figure 6, it is shown that the entrance area of the wage hall had an increased surface density compared to the rest of the point clouds exhibited. The point cloud in the roof area of the wage hall was in the value range of the average surface density. In the right part of the front facade, an increased surface density could be seen. In contrast, the left part of the front facade showed lower surface densities and missing data points. Overall, increased surface densities can be seen in the front facade and entrance area of the wage hall.

### 3.2 Cloud-To-Cloud Distance

**FIGURE 7:**
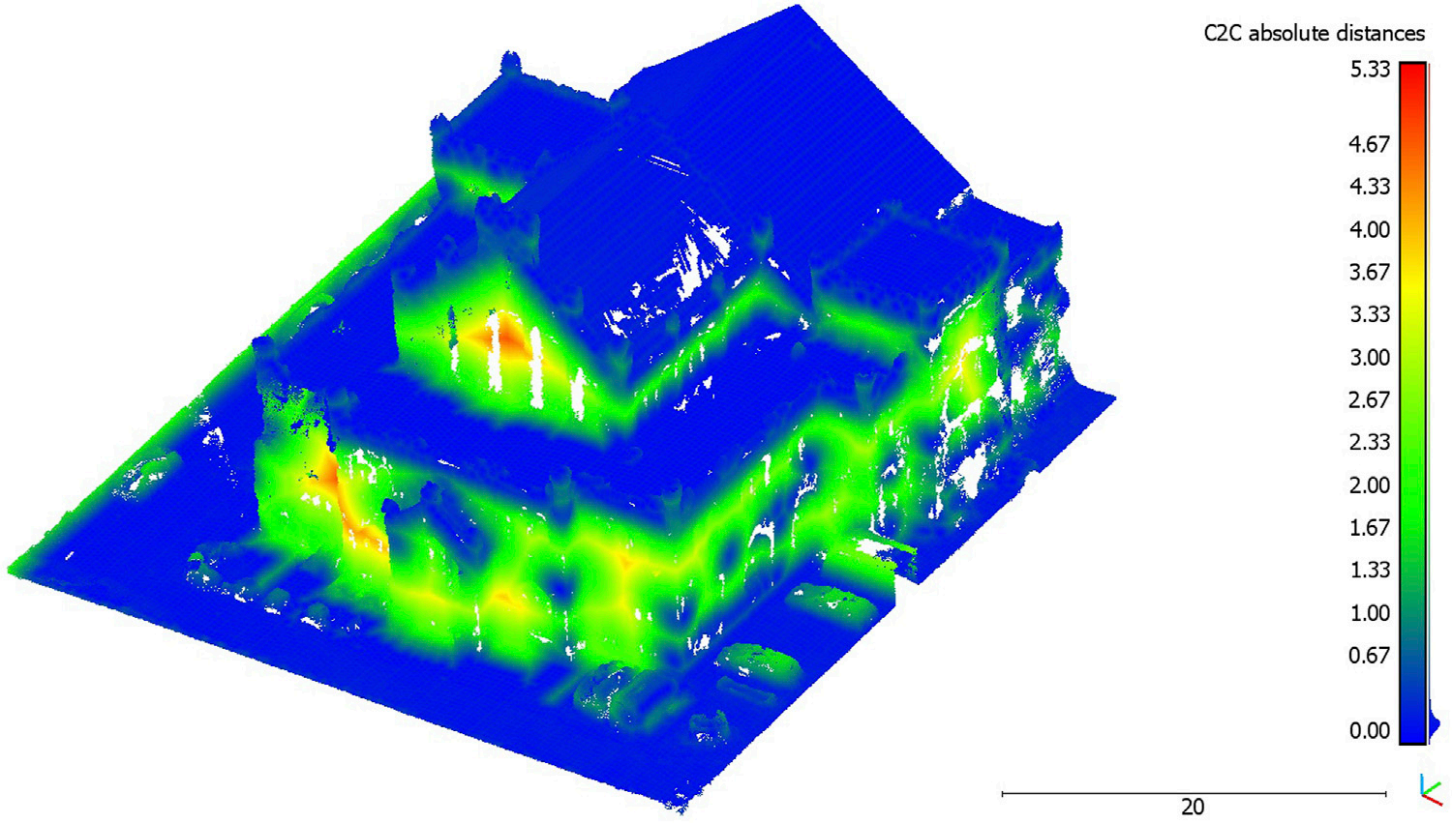
Cloud-to-cloud distance between ALS and UAV point clouds visualized on the UAV point cloud.

**FIGURE 8:**
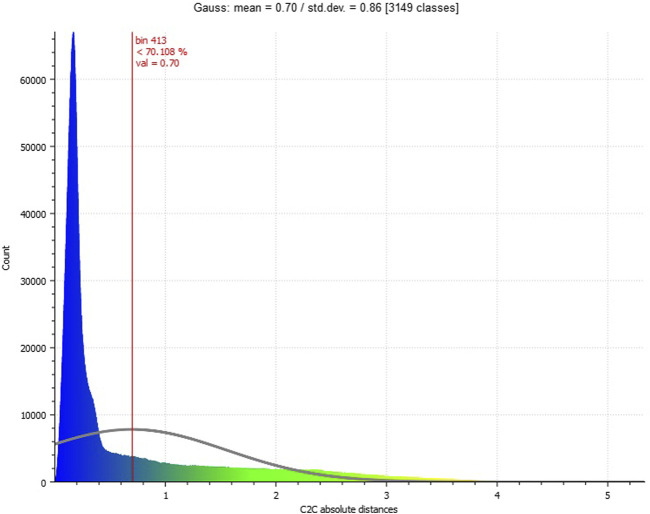
Histogram on cloud-to-cloud distance distribution (between ALS and UAV point clouds).

The result of the cloud-to-cloud distance is shown in Table 2. It depicts the average distance of each point of the ALS point cloud to each point of the UAV point cloud in a search radius of 0.15 m, including the standard deviation. When looking at the visualization of the cloud-to-cloud distance in Figure 7, the UAV point cloud of the data collection of the wage hall showed increased distances in the area of the facades of the building. The increased distances in the facade areas may be related to the fact that the surface density of the ALS point cloud showed a low density and coverage of data points in the area of the facade. It is noticeable that low distances prevail in the area of the facades, in which data points exist in the facade area of the ALS point cloud. In the roof and floor areas, small distances can be seen when looking at Figure 7. These are areas that are covered by data points in both point clouds. Moreover, the value distribution (histogram) of the cloud-to-cloud distance (Figure 8) shows that 70.1% of the distances are below the mean distance of 0.7 m.

**TABLE 2:** Cloud-to-cloud distance of UAV point clouds to the ALS point cloud (wage hall flight campaign).

Cloud-to-cloud distance ø (m)	σ (m)
0.7	0.86

### 3.3 Cloud-To-Mesh Distance

**FIGURE 9:**
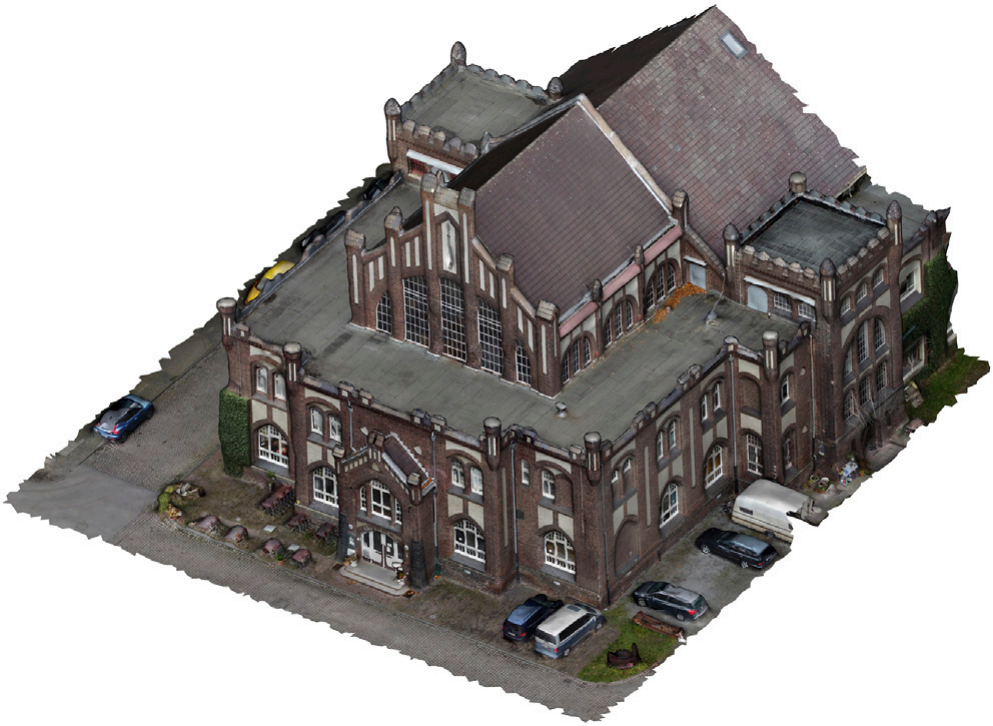
3D reconstruction of the wage hall based on the microdrone (DJI Mavic Mini) flight.

The visual impression of the 3D reconstruction of the wage hall, which serves as the data basis for the cloud-to-mesh distance, can be seen in Figure 9. In terms of appearance, the old wage hall and its (partly vegetated) historical facades are well represented in the 3D reconstruction. Around the building, reconstructions of additional objects, such as cars, can also be visually identified.

**FIGURE 10:**
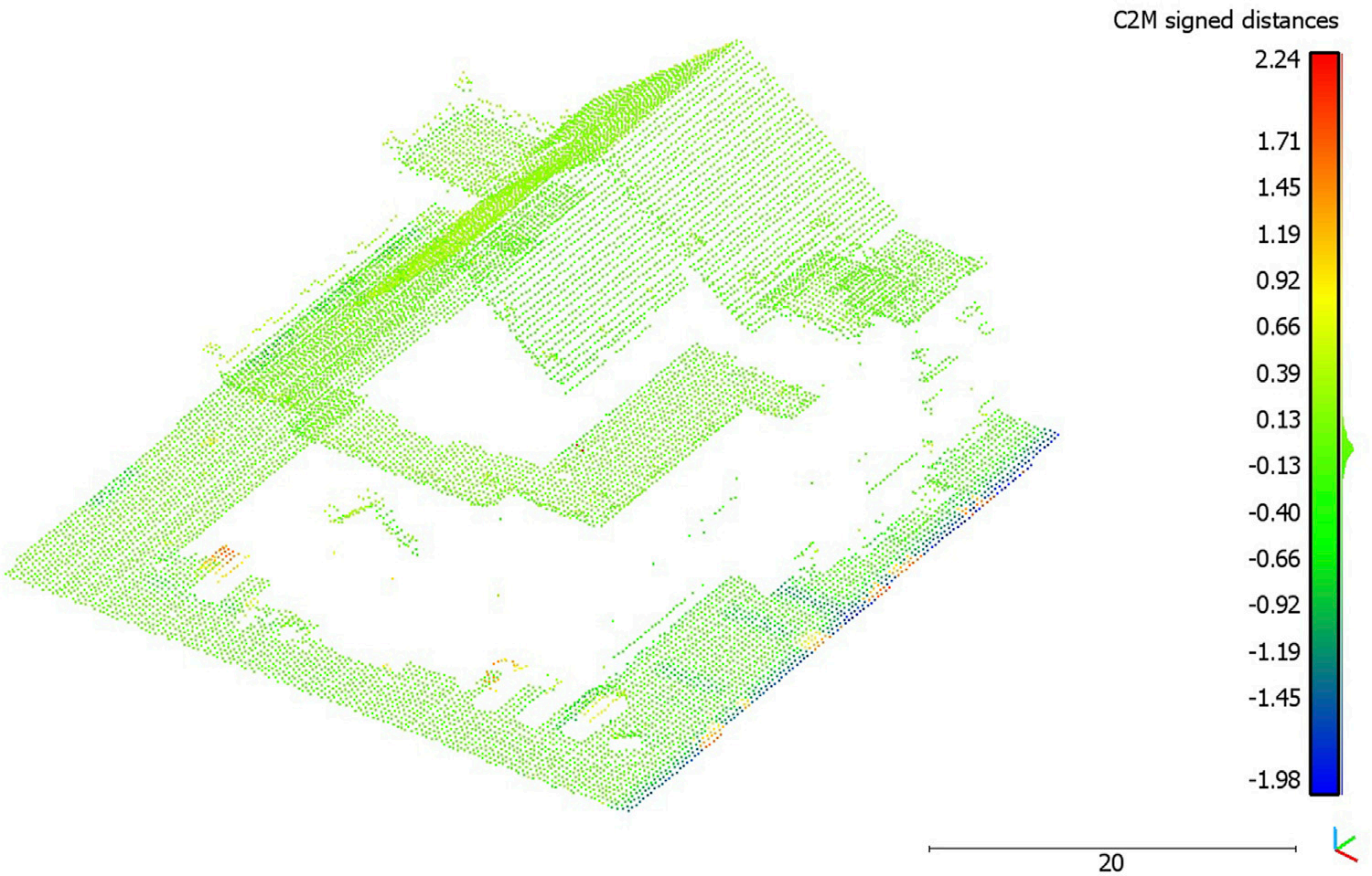
Visualized cloud-to-mesh distance.

**FIGURE 11:**
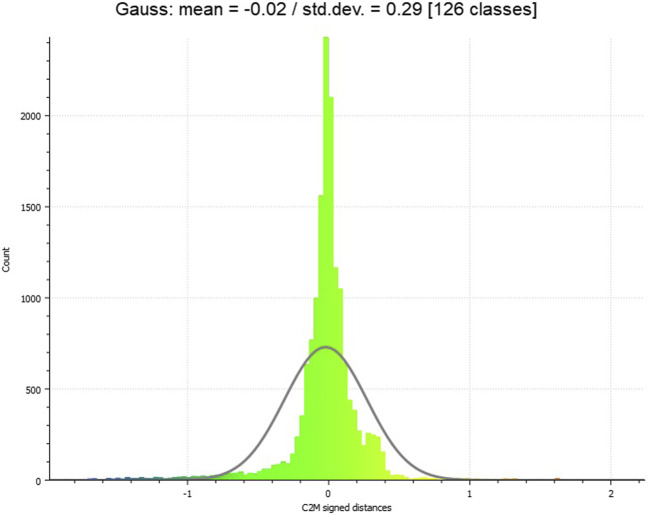
Histogram on cloud-to-mesh distance distribution. The microdrone-based 3D model within an immersive virtual environment.

In Table 3, the result of the cloud-to-mesh distance is given. The visualized result of the cloud-to-mesh distance approach is presented in Figure 10. For the 3D reconstruction of the wage hall to the ALS point cloud, a mean distance of −0.02 m resulted and had a standard deviation of 0.29 m. Increased deviations in the eastern edge area of the investigation area could be determined. This could be caused by a partly inaccurate geo-referencing of the UAV point cloud, which caused a non-uniform comparison surface during the adjustment of the point clouds to the investigation area. In addition, increased distances can be seen at locations that can be recognized as representing vehicles, within the ALS point cloud. When examining the 3D reconstruction in these areas, it is noticeable that there are no vehicles there. As the distance of each point of the point cloud to the polygon mesh is calculated in the cloud-to-mesh distance, the missing data coverage of the facades in the UAV point cloud is omitted here, which was also the case within the cloud-to-cloud distance computing (Section 3.2). The distances from the points in the point cloud are, thus, only calculated for the locations where data points are also available. The missing data points for the building facade are, thus, not included in the distance calculation. When inspecting the roof and floor areas in Figure 10, small distances can be seen here, which is also represented by the data distribution in the data histogram (Figure 11).

**TABLE 3:** Cloud-to-mesh distance (wage hall flight campaign).

Signed distance ø (m)	σ (m)
−0.02	0.29

Unity Engine supports 3D models with the file formats Filmbox (.fbx), COLLADA (.dae), Drawing Interchange File Format (.dxf) and Wavefront OBJ (.obj) (Unity Technologies 2022). For technical use in Unity, the 3D model created in the workflow (in Wavefront OBJ file format) does not require any conversion steps and can, thus, be imported directly into the game engine.

The potential of representing photogrammetric 3D records of cultural heritage sites by the modern possibilities of freely accessible game engines and compatible virtual reality systems has been addressed in several recent studies (e.g., Büyüksalih et al., 2020; Kersten et al., 2021; Koutsoudis et al., 2007). The possibilities of low-budget ultra-lightweight UAVs, such as the microdrone DJI Mavic Mini, for deriving and creating immersive virtual environments have only hardly been explored so far.

**FIGURE 12:**
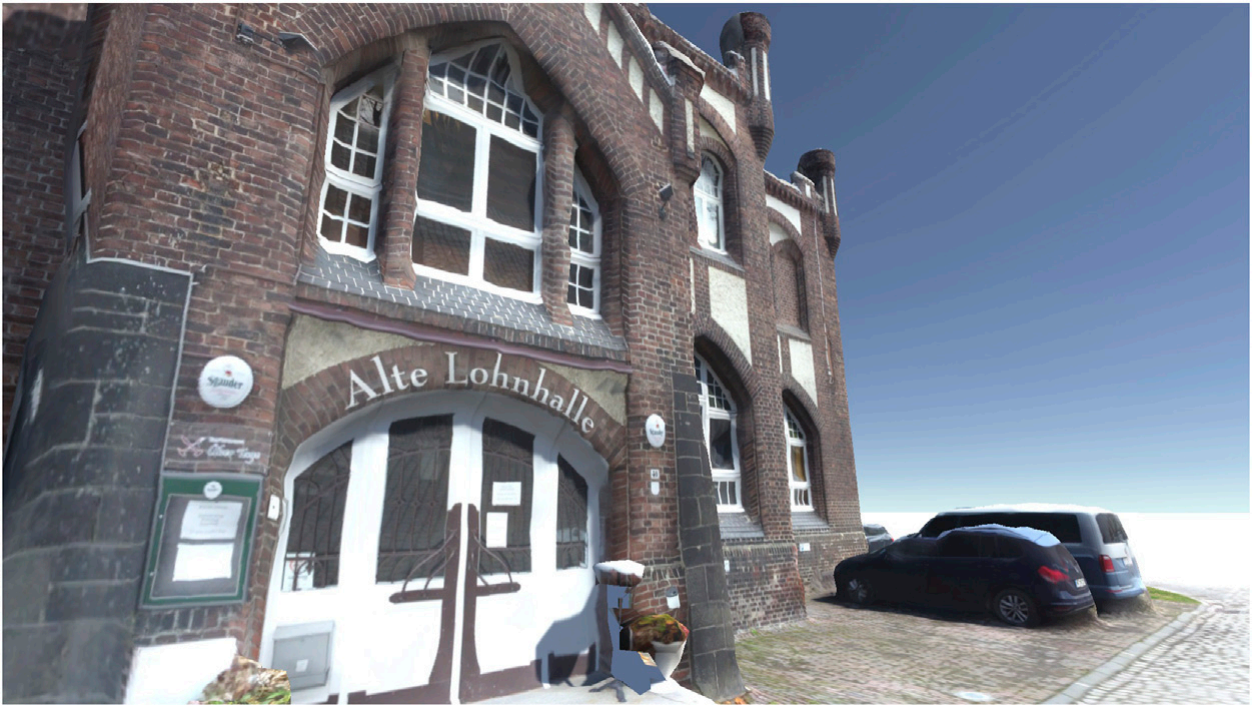
View on the front facade of the historical wage hall in the game engine Unity.

Figure 12 gives an impression of the entrance facade of the case example (historical wage hall of a Ruhr area coal-mining site, today used as a restaurant). The view is taken ‘through the virtual eyes’ of the avatar. In this image, the quality is indicated: The complex facade including different ornaments is represented. Moreover, the microdrone-based acquisition of the 3D data and image (RGB) textures facilitate detailed representations. The figure indicates that textual ornaments on the facade in different sizes can be read, like the name of the building (“Alte Lohnhalle”) and the name of a local brewery (“Stauder”). Moreover, a menu card is indicated. The figure also indicates limitations in the final model, leading to slightly unrealistic impressions, which might also affect the user’s feeling of immersion. For example, the shifted window above the entrance is distorted as the 3D point cloud did not consider the offset. Additional objects, such as the parking cars or flowerpots, however, indicate good quality of the surrounding objects of the building.

## 4 Discussion

The data processing was based on open-source software solutions. The measures used to analyze this performance included two approaches: the cloud-to-cloud distance (comparative analysis of the point cloud data accuracy) and cloud-to-mesh distance (comparative analysis of the 3D reconstruction based on the point cloud data).

The analyses based on the cloud-to-cloud distance measure indicate the potentials of low-budget microdrones for highly accurate 3D point clouds. Comparing these data with professional official datasets (open geo-spatial data), the microdrone-based approach leads to a very similar data quality in terms of the spatial accuracy (Figure 7). The data value distribution (Figure 8) points to a concentration of more than 70 percent of the acquired data lower than the mean value (0.7 m, Table 2). Higher distances are caused by a lack of data availability in the officially offered ALS data as their acquisition does not cover data on building facades. The flexibility of easy-to-fly microdrones in terms of individualization of data acquisition points (Figure 2) not only fills data gaps regarding building facades but also facilitates an increase in acquisition points and, subsequently, the (surface) density of point clouds (Figure 6). This can contribute to a strengthening of data accuracy of, for example, complex (historical) facades, which is in line with previous studies on drone-based 3D reconstruction of cultural heritage sites (Febro 2017; Adami et al., 2019; Bakirman et al., 2020).

The results of the cloud-to-cloud distance analysis are underlined by the cloud-to-mesh distance analysis. The mean (signed) distance value (−0.02 m, Table 3) and its low standard deviation (0.29, Figure 11) represent a high accuracy of the 3D reconstruction. The 3D reconstruction of the meshed and textured model of the wage hall (Figure 9) gives a visual impression of the results and underlines the accuracy. This 3D model, originating in the microdrone-based flight within this exploratory study, can further be imported into a game engine, supporting the experience of an immersive virtual environment of the represented post-industrial landmark.

Due to the low number of data points in the ALS point cloud (Table 1), it would be interesting to evaluate and compare other professional-generated point clouds to the UAV point cloud. The focus could be on the comparison of the building facades. In this approach, the representation of the facade by the UAV point cloud predominated in terms of available data points in the facade. A direct comparison of the facade with the ALS point cloud could not be explored in this study caused by the lack of data coverage (Figure 5).

## 5 Summary and Outlook

This exploratory study points to the potential that photorealistic 3D models can be generated by using low-cost microdrone images in combination with freely available software. The point clouds derived from the acquired drone data were of a spatial accuracy similar to official airborne laser scanning (ALS) data sets provided by governmental open data initiatives in North Rhine–Westphalia, Germany. The used measures, cloud-to-cloud distance, and cloud-to-mesh distance, also pointed to an advantage of the microdrone-based approach over the ALS data sets, when it comes to questions of applying image textures and representing building facades. This is also represented in the data on the surface density. In this study, the number of GCPs and their distribution were not considered, but they should be included as an additional component in future approaches [cf. Balletti et al. (2015)].

This study provides a workflow that is based on a low-budget data acquisition approach and on a workflow of data processing built on open-source software solutions. It allows various users, including representatives of citizen science, to create and evaluate 3D reconstructions of an individual area of interest. This workflow may be applied by non-experts to create individualized and flexible image-textured 3D models. It may also animate to explore and exploit the full potential of mass media photography equipment that goes beyond traditional 2D photography of places of interest, such as post-industrial landmarks. In future research studies, the suitability of the detailed point clouds and (immersive) 3D models should be further explored for specific application purposes in spatial disciplines. This research should particularly include user feedback and empirical data on (cognitively) processing these 3D applications. For future studies, it could also be a valuable extension to use other microdrones equipped with RT/PPK capable receivers to further improve the data accuracy.

## Data Availability

The raw data supporting the conclusions of this article will be made available by the authors, without undue reservation.
